# Effectiveness of Nola Pender’s Health Promotion Model: A Comprehensive Approach for Enhancing Healthy Behaviors and Quality of Life in Adults

**DOI:** 10.3390/ijerph22101506

**Published:** 2025-09-30

**Authors:** Indiana L. Rojas-Torres, Claudia M. Garizábalo Dávila, Keidis S. Ruidíaz Gómez, Shirley P. Fernández Aragón, Diana M. Perea-Rojas, Greys M. Rodelo Olmos, Norma I. Liñán Meléndez

**Affiliations:** 1 Faculty of Health Sciences, Universidad Simón Bolívar, Barranquilla 080002, Colombianorma.linan@unisimon.edu.co (N.I.L.M.); 2 Department of Health Sciences, Universidad de la Costa, Barranquilla 080002, Colombia; cgarizab2@cuc.edu.co; 3 Department of Nursing, Faculty of Health Sciences, University of Sinú, Cartagena 130001, Colombia; 4 Faculty of Nursing, Universidad de Cartagena, Cartagena 130001, Colombia; sfernandeza@unicartagena.edu.co

**Keywords:** health promotion, physical activity, community health, health behavior

## Abstract

Health behavior encompasses the attitudes, decisions, and actions that individuals adopt in their daily lives and that directly influence their well-being. The objective of this study was to evaluate the effectiveness of a nursing intervention based on Nola Pender’s Health Promotion Model in promoting healthy behaviors among adults over 20 years of age. A quasi-experimental design was used with a sample of adults from a Colombian community. In 2022, sociodemographic data were collected, and the Health-Promoting Lifestyle Profile II (HPLP-II), developed by Nola Pender to measure healthy lifestyles, was administered. In 2023, the intervention was carried out in 24 group sessions of 40 min each, supported by printed educational material and focused on the dimensions of Pender’s theory. Statistical analysis using nonparametric tests such as the Mann–Whitney U and Kruskal–Wallis tests showed significant improvements: the overall score increased from 22.92 in the pre-test to 27.30 in the post-test. The physical activity dimension received the lowest score, while the spiritual growth dimension showed the highest improvement. The results indicate that nursing interventions based on Pender’s model led to statistically significant improvements in multiple dimensions of healthy behavior, with the greatest impact on spiritual growth and health responsibility.

## 1. Introduction

The term health behavior refers to the way in which people integrate their environment with their lifestyle, a relationship influenced by behavioral factors such as habits and routines, as well as individual motivational factors such as goals and values [1]. According to Álvarez and Luz [2], these behaviors are expressed through responses learned during the socialization process, giving rise to patterns that reflect life choices shaped by environmental and social determinants of health.

The proper adoption of these healthy habits supports satisfactory levels of well-being; however, poor practices can have negative repercussions for health [3]. Research has shown that risky behaviors, such as alcohol consumption, smoking, unhealthy diets, physical inactivity, and uncontrolled stress, are closely associated with the development of non-communicable diseases (NCDs), posing a significant threat to health and quality of life [4,5].

According to the Pan American Health Organization [6], NCDs cause 41 million deaths annually, representing 71% of all global deaths. Each year, around 15 million people between the ages of 30 and 69 die from NCDs, with more than 85% of these deaths occurring in low- and middle-income countries. Cardiovascular diseases (17.9 million), cancer (9.0 million), respiratory diseases (3.9 million), and diabetes (1.6 million) account for the majority of this burden.

In addition to their clinical impact, these diseases generate a considerable economic burden due to medical costs and lost productivity. In this context, health promotion and maintenance are key strategies for reducing the incidence of NCDs and promoting long-term well-being [7].

For this reason, the Health Promotion Model (HPM), proposed by Nola Pender, was employed. It emerged as an integrative perspective within nursing theories, focusing on the promotion, encouragement, and understanding of healthy habits [8]. This model, which is widely used in community settings, helps identify factors that influence health decisions and guides individuals toward positive behaviors [9].

Unlike other behavioral models, HPM is not limited to disease prevention. Instead, it integrates personal, motivational, and interpersonal factors that influence the adoption of healthy lifestyles. Its holistic perspective aligns with the nursing discipline’s goal of promoting physical, emotional, and social well-being.

As suggested in the literature, Pender’s model facilitates effective health management through educational and nursing interventions, contributing to the self-care process by promoting healthy eating, physical activity, motivation, and self-esteem, among other aspects [10,11]. One of the model’s main strengths is its preventive approach, enabling intervention before complications compromise well-being. Its application is particularly effective in community contexts, where its benefits extend not only to individuals but also to their families and social circles [12,13].

The objective of this study was to assess the effectiveness of an educational intervention based on Nola Pender’s model in promoting healthy behaviors among adults in a Colombian community.

## 2. Materials and Methods

### 2.1. Design and Sample

A quasi-experimental study was conducted with a single group, and pre- and post-intervention measurements in a community in the city of Barranquilla, Colombia. The objective was to evaluate the effects of an educational intervention on healthy lifestyles in adults over 20 years old, based on the dimensions proposed in Nola Pender’s Health Promotion Model.

The population consisted of 165 individuals who participated in a community.

The intervention project aimed at promoting healthy habits. The sample was selected through convenience sampling, in collaboration with community leaders from the intervention area, who extended invitations to potential participants. Those who expressed interest were assessed according to previously established inclusion criteria. This strategy was chosen for practical and operational considerations within the community context, as it allowed direct access to available and willing individuals during the implementation period. Although this type of sampling limits the generalizability of the results, it is appropriate for intervention studies in real-world contexts where the primary objective is to assess the applicability of educational strategies in specific settings.

The sample size was calculated based on the parameters of a pre–post design: 95% confidence level, 90% power, a minimum expected difference of 0.4 units, and a standard deviation of 1.1. The base sample size was estimated at 79 participants, later adjusted for a 20% attrition rate, resulting in a minimum requirement of 99 individuals.

Of the 165 individuals initially invited, 13 declined to participate, 11 did not attend all the activities of the community intervention, and 7 did not fully complete the questionnaires.

As a result, 117 participants were included in the final analysis, which reinforced the statistical power and the validity of the results. From an ethical standpoint, the objective of the study was explained to each participant, inclusion criteria were verified, written informed consent was obtained, and participants were formally registered in the project.

### 2.2. Measurement

During 2022, sociodemographic data were collected, and the Health Promoting Lifestyle Profile II (HPLP-II), developed by Nola Pender to measure healthy lifestyles, was applied. In 2023, the intervention was implemented, consisting of 24 group sessions lasting 40 min each, supported by printed educational materials and focused on the dimensions of Pender’s theory.

Home visits were conducted in 2022 to administer the pre-test questionnaire, which included sociodemographic variables such as age, gender, occupation, educational level, and affiliation with the General Social Security Health System (SGSSS), as well as variables from the Spanish version of the Health Promoting Lifestyle Profile II (HPLP-II) for adults by Nola Pender. This instrument includes 52 items divided into six dimensions: (1) health responsibility, related to interest in personal well-being, shown through behaviors such as seeking medical attention for unusual symptoms or seeking information to improve health; (2) physical activity, assessing the frequency and willingness to engage in physical exercise; (3) nutrition, evaluating participants’ dietary behaviors, including frequency, quantity, and quality of food intake; (4) spiritual growth, associated with the search for meaning and purpose in life, the development of personal goals, inner peace, and connection with a higher power; (5) interpersonal relationships, referring to the individual’s willingness and ability to socialize and communicate assertively; and (6) stress management, exploring symptoms of anxiety, sleep habits, recreational activities, and relaxation techniques. Behaviors were rated on a Likert scale from 1 to 4, where 1 = Never, 2 = Sometimes, 3 = Often, and 4 = Routinely.

This instrument, derived from the original version of the HPLP-II, reported Cronbach’s alpha coefficient of 0.94, and for the six subscales, values ranged from 0.79 to 0.87. It was also used in a study with Colombian women, where a variance of 45.9% was recorded, reliability ranged from 0.7 to 0.9, and a Cronbach’s alpha of 0.93 was reported [14].

### 2.3. Data Analysis Strategy

Based on the results obtained, an intervention plan was designed and implemented during 2023, divided into two academic semesters. The first semester took place from February to May, and the second from August to November. A total of 24 sessions were conducted—12 per semester—focused on educational interventions aligned with each dimension of the model. Each session lasted 45 min, was structured in phases (introduction, development, and evaluation), and utilized printed educational materials (Table 1).

After the implementation of the educational program, the same instrument used in the diagnostic phase was administered again. Data were analyzed using SPSS version 20, and the meaning was calculated to summarize the values of the general scale and each specific dimension. Non-parametric tests, such as the Mann–Whitney U and Kruskal–Wallis tests, were used to compare group ranks in the bivariate analysis between HPLP-II dimensions and sociodemographic variables. The level of statistical significance was set at *p* < 0.05.

## 3. Results

Of the total participants, 25% (n = 29) were between 20 and 25 years old, 60% (n = 70) were women, 41% (n = 48) identified as housewives, 43% (n = 50) had completed high school, and 72% (n = 84) were affiliated with the subsidized regime of the General System of Social Security in Health (SGSSS) (Table 2).

Regarding the results obtained by summing the Likert scale categories of the HPLP II items, these are detailed in Table 3. A significant increase was observed in the average scores of all dimensions evaluated in the post-test.

**Figure 1:**
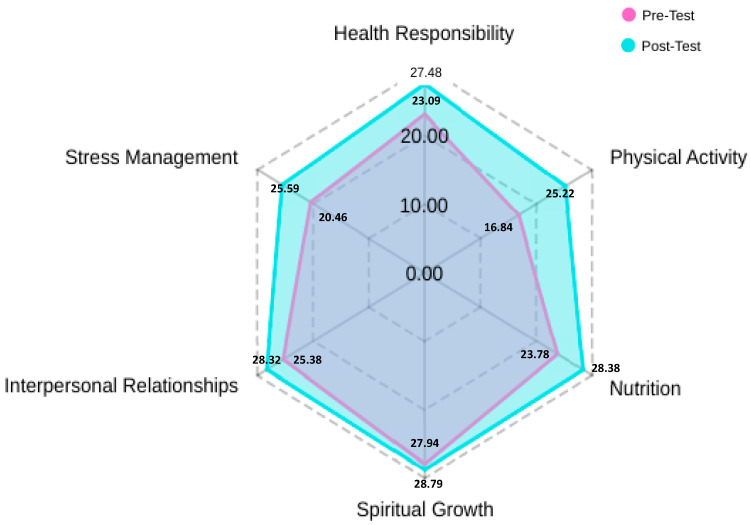
Average Scores by Dimension: Pre-Test vs. Post-Test.

The pre-test results showed initial values ranging from 16.8 to 27.94. The physical activity dimension had the lowest average, while spiritual growth had the highest average. In the post-test, the results show an overall average ranging from 25.22 to 28.79. Although the physical activity dimension had the lowest proportion in this evaluation, the interventions were effective, reflecting an 8.38 increase in results. In general terms, there is an average increase of 0.85 to 8.38 in the specific dimensions: health responsibility (4.39), physical activity (8.38), nutrition (4.6), spiritual growth (0.85), interpersonal relationships (2.94), and stress management (5.13). (See Figure 1).

Table 4 presents the dimensions of a healthy lifestyle that showed statistically significant differences (*p* < 0.05) according to sociodemographic variables, both in the pre-test and post-test. In the pre-test, significant associations were identified between the health responsibility dimension and the variables age (*p* = 0.039) and sex (*p* = 0.035). The spiritual growth dimension showed differences according to age (*p* = 0.030), occupation (*p* = 0.006), and SGSSS affiliation regime (*p* = 0.007), while interpersonal relations were associated with the affiliation regime (*p* = 0.020). In the post-test, age remained associated with health responsibility (*p* = 0.015) and emerged as a significant variable in physical activity (*p* = 0.001) and stress management (*p* = 0.003). Additionally, spiritual growth and interpersonal relations maintained their association with the affiliation regime (*p* = 0.040). The directions of these differences are not interpreted, as the mean scores by category were not specified. These findings reflect the existence of differential patterns in the healthy lifestyle dimensions based on key sociodemographic characteristics, before and after the intervention.

On the other hand, it is important to highlight that, based on the responses to the Health Promoting Lifestyle Profile II, key behaviors were identified that allow for the assessment of participants’ lifestyles from a clinically meaningful perspective. According to the questions asked, a low frequency was observed in practices such as checking one’s pulse during physical activity, attending health education programs, and performing relaxation or meditation techniques. This indicates weaknesses in the dimensions of physical activity, health responsibility, and stress management. In contrast, favorable behaviors were reported, such as greater awareness of what is important in life, recognition of others’ achievements, and the habit of eating breakfast, reflecting strengths in the dimensions of spiritual growth, interpersonal support, and nutrition. (See Supplementary Materials).

## 4. Discussion

The results obtained show that certain sociodemographic variables, particularly age, play a determining role in the variability of healthy lifestyles, both before and after an educational intervention. Age was significantly associated with various dimensions, including health responsibility, physical activity, spiritual growth, and stress management, suggesting that the life cycle may influence the adoption of self-care practices and the willingness to change health-related behaviors.

On the other hand, a positive increase was observed in all of Pender’s proposed dimensions following the interventions evaluated in the post-test. Similar studies have also demonstrated improvements in promoting healthy habits, especially in areas such as nutrition, physical activity, stress management, adherence to medical treatments, and among patients with chronic diseases [15,16,17].

Specifically, the findings showed that the physical activity dimension had the lowest average. Various studies agree that the main obstacles to changing healthy habits related to physical activity are psychological, social, economic, and environmental factors. The most relevant include lack of time due to work and family responsibilities, low personal motivation to maintain exercise routines, and the absence of safe and adequate spaces for physical activity. Social support and the perception that physical activity is not a priority also significantly influence adherence. Therefore, it is crucial to address these factors by providing a supportive environment and adequate resources, such as motivation, self-confidence, family support, and a favorable situational context [18,19].

Conversely, the spiritual growth dimension reached the highest average. This result could be explained by the community’s sociocultural characteristics, where group reflection activities, experience-sharing, and interpersonal support contributed to the construction of a personal meaning oriented toward overall well-being. These results are consistent with those of Sánchez and Arias [20], who demonstrated the effectiveness of interventions aimed at promoting mental well-being, a sense of peace, and connectedness. Similarly, a study conducted in a community of older adults in China found that social support significantly predicted positive changes in spiritual growth and health responsibility during health promotion activities [21]. Another study in a community-based adult population showed that spiritual growth, stress management, and interpersonal relationships explained up to 53% of the variability in psychological well-being and life satisfaction [22]. Additionally, the bivariate analysis showed that this dimension holds significant relevance among individuals affiliated with the subsidized health insurance regime.

Regarding the health responsibility dimension, bivariate analysis revealed the highest scores in both the pre-test and post-test, especially among women aged 41 to 45. A study conducted in Peru with university adolescents also showed positive results in improving exercise, health responsibility, and stress management by focusing on motivational strategies during the sessions [15,23].

Although no statistically significant relevance was found in the nutritional dimension, the results reflected a positive response from the study participants. Authors Peraza, Benítez, and Galeano [24] proposed a similar strategy to improve an institutional nutritional guidance program aimed at a specific academic community. The analysis indicated that nutritional disorders were prevalent among both students and staff. Other similar studies have demonstrated the effectiveness of Pender’s model, as its components enabled the identification and effective management of nutritional habits [25]. This finding facilitates the formulation of appropriate educational intervention programs from a nursing perspective.

Finally, regarding stress management, post-test results showed high scores among individuals aged 26 to 30. These results confirm that stress has become one of the most common problems worldwide. There is a wide range of techniques available to address the various causes of stress. A similar study states that each person, group, or institution must select the most appropriate techniques based on the type of risk to which they are exposed, the nature of the stress they experience, and their personal and organizational characteristics [20].

The intervention based on Nola Pender’s Health Promotion Model proved effective in improving all the dimensions evaluated. These results suggest that Pender’s model is a valuable tool to guide interventions that promote healthy habits and improve quality of life [26]. Continuing to apply strategies based on this model could help maintain and strengthen the progress achieved, promoting a healthier and more empowered population.

## 5. Conclusions

The educational intervention based on Nola Pender’s Health Promotion Model was associated with positive changes in the adoption of healthy lifestyles among the participants. The results confirm that this approach provides a solid foundation for designing nursing strategies focused on physical, emotional, and social well-being.

These findings highlight the importance of considering sociodemographic variables when designing and evaluating health interventions, especially those aimed at promoting healthy lifestyles. In particular, the need to implement differentiated and culturally sensitive strategies that respond to the characteristics and needs of various population groups is suggested.

Although the physical activity dimension showed the lowest values, a significant improvement was achieved, reflecting the model’s potential to address even the most resistant behaviors to change. Likewise, spiritual growth and health responsibility stood out as strengths of the intervention. These findings support the usefulness of the Pender model as a fundamental tool for community work, enabling nursing professionals to plan, implement, and evaluate effective actions that promote healthy lifestyles and improve quality of life, especially in contexts of high social vulnerability.

## 6. Limitations

One of the main limitations of this study was the use of convenience sampling. Although this type of sampling allows direct access to the target population and facilitates the implementation of interventions, it may limit the ability to generalize the results to other populations with different characteristics. Nevertheless, this choice was justified by the nature of the study design and the need to work with a specific population that met the criteria, which provided valuable findings and insights for the context in which the research was conducted.

Another relevant limitation is the possibility of selection bias resulting from the participant recruitment process, which was conducted with the support of community leaders. While this strategy facilitated outreach and acceptance of the intervention in a socially complex context, it may have favored the inclusion of individuals with greater motivation, availability, or commitment to health care, which could have positively influenced the observed results, regardless of the intervention itself. This potential self-selection limits the generalizability of the findings to the entire community and requires caution when interpreting effects attributed exclusively to the implemented strategy.

It is acknowledged that using a pre-test–post-test design without a control group limits the ability to establish causal relationships. However, this approach was adopted due to the particular conditions of the context, which made the implementation of an additional control group unfeasible. Social, environmental, and security-related factors in the community prevented the application of a conventional experimental design, leading to the use of a quasi-experimental design with a single group. These aspects are explicitly recognized as limitations of the study.

## Figures and Tables

**Table 1:** Planning of educational interventions.

Dimension	Objective	Intervention
Health Responsibility	Promote interest in personal well-being, manifested through behaviors such as proactively seeking medical care and relevant information to improve health.	Educational workshops on: prevention and management of non-communicable diseases: diabetes, hypertension, respiratory infections, adherence to treatments
		Screening sessions
		Comprehensive health campaigns
		Awareness campaigns using social media and Q&A sessions.
Physical Activity	Encourage participation in physical activities, promoting the importance of exercise for health.	Group exercise and stretching sessions
		Dance sessions
Nutrition	Promote the adoption of balanced eating habits, encouraging regular consumption of nutritious foods and moderation in the intake of saturated fats, sugars, and processed foods.	Educational workshops on: food labeling, types of food, portions, and nutritional contributions
		“I choose my food” game, healthy recipes, nutritional Q&A game
		Nutritional assessment and counseling
		Dramatization on types of food and their contributions
Spiritual Growth	Stimulate spiritual growth and the establishment of short, medium, and long-term goals.	Recreational activities: self-assessment, mandala drawing, motivational reading with group reflections, shared collage with images of spiritual growth
		Motivational video and reflection on its message
Interpersonal Relationships	Facilitate the development of skills to establish healthy interpersonal relationships.	Educational workshops on healthy coexistence followed by dramatization activities to enrich the experience
		Recreational activities through role-playing games and teamwork
Stress Management	Develop adaptive strategies that allow individuals to cope with daily stress in a healthy and constructive manner.	Mindfulness activities: breathing exercises to reduce physical and mental tension, guided meditation, and sensory exploration exercises.

**Table 2:** Sociodemographic variables.

		Number n	%
Age (years)	20–25	29	25%
	26–30	18	15%
	31–35	8	7%
	36–40	18	15%
	41–45	13	11%
	46–50	22	19%
	>50	9	4%
Sex	Male	47	40%
	Female	70	60%
Employment	Housewife	48	41%
	Formal job	46	39%
	Informal job	7	6%
	Unemployed	2	1%
	Student	13	12%
Education level	Middle school	20	17%
	High School	50	43%
	Technical	14	12%
	University	4	3%
	No studies	7	6%
	Does not report	22	19%
Health Insurance System	Employer-Sponsored	29	25%
	Subsidized	84	72%
	Not affiliated	4	3%
TOTAL		117	100%

**Table 3:** Dimensions of the Health Promotion Life Profile II Scale by Pender.

Dimensions	Pre-Test					Post-Test					*p*-Value
	Overall Average	Never (Average)	Sometimes (Average)	Frequently (Average)	Routinely (Average)	Overall Average	Never (Average)	Sometimes (Average)	Frequently (Average)	Routinely (Average)	
Health Responsibility	23.09	18.3	20.2	24.5	28.1	27.48	15	17	32	53	<0.01 *
Physical Activity	16.84	14.2	16.5	21.2	25.4	25.22	13	13	37	55	<0.01 *
Nutrition	23.78	19.5	20.6	25	27.7	28.38	12	14	36	55	<0.01 *
Spiritual Growth	27.94	23.6	23.7	27.4	31	28.79	8	14	42	53	<0.01 *
Interpersonal Relationships	25.38	20.3	22.2	25.9	28.9	28.32	9	14	44	50	<0.01 *
Stress Management	20.46	17.9	18.2	20.9	24.4	25.59	9	12	42	54	<0.01 *

* Statistically Significant.

**Table 4:** Dimensions of the Health Promotion Life Profile II Scale by Pender.

Dimensions	Variable Sociodemographic	*p*-Value	
		Pre-Test	Post-Test
Health Responsibility	Age	0.039 *	0.015 *
	Sex	0.035 *	—
Physical Activity	Age	—	0.001 *
Nutrition	Age	0.030	—
	Occupation	0.006 *	—
	Health Insurance Regime (SGSSS)	0.007 *	0.040 *
Spiritual Growth	Health Insurance Regime (SGSSS)	0.020 *	—
Stress Management	Age	—	0.003 *

* Statistically Significant.

## Data Availability

The original contributions presented in this study are included in the article. Further inquiries can be directed to the corresponding author (s).

## Supplementary Materials

The following supporting information can be downloaded at: https://doi.org/10.5281/zenodo.15285479 (accessed on 14 May 2025).

## Abbreviations

The following abbreviations are used in this manuscript:

HPLP-II	Health Promoting Life Profile II
NCDs	Non-communicable diseases
HPM	Health Promotion Model
